# Supplementary material for: Silver Nanostructured Substrates in LDI-MS of Low Molecular Weight Compounds
Source: Materials (Basel). 2022 Jul 2;15(13):4660. doi: 10.3390/ma15134660 (PMC9267646; doi:10.3390/ma15134660)
Supplement: Supplementary file 1 [file materials-15-04660-s001.zip › materials-1756760-supplementary.pdf]

Supplementary material for manuscript entitled 'Silver nanostructured substrates in LDI-MS of low molecular weight compounds'

by Gulyaim Sagandykova, Piotr Piszczek, Aleksandra Radtke, Radik Mametov, Oleksandra Pryshchepa, Dorota Gabryś, Mateusz Kolankowski and Paweł Pomastowski

Table S1. The *m/z* values for compounds detected using LDI-MS nanostructured substrates

Compound	*m/z*	[M]
plate AgPs0.02		
**14:0-13:0-14:0 TG**	820.13	$[M+^{107}Ag]^+$
**14:0-15:1-14:0 TG**	848.35	$[M+^{109}Ag]^+$
**14:0-17:1-14:0 TG**	874.54	$[M+^{107}Ag]^+$
**16:0-15:1-16:0 TG**	904.63	$[M+^{109}Ag]^+$
**16:0-17:1-16:0 TG**	932.88	$[M+^{109}Ag]^+$
**16:0-19:2-16:0 TG**	958.10	$[M+^{109}Ag]^+$
**18:1-17:1-18:1 TG**	876.57	$[M+H]^+$
**18:1-19:2-18:1 TG**	902.78	$[M+H]^+$
**adonitol**	258.98	$[M+^{107}Ag]^+$
**cholesterol**	493.41	$[M+^{107}Ag]^+$
**alanine**	195.96	$[M+^{107}Ag]^+$
**fructose**	286.97	$[M+^{107}Ag]^+$
**glucose**	286.96	$[M+^{107}Ag]^+$
**methionine**	255.97	$[M+^{107}Ag]^+$
**palmitic acid**	363.14	$[M+^{107}Ag]^+$
**oleic acid**	389.16	$[M+^{107}Ag]^+$
**phenylalanine**	271.99	$[M+^{107}Ag]^+$
**serine**	211.59	$[M+^{107}Ag]^+$
**shikimic acid**	281.00	$[M+^{107}Ag]^+$
**17:0-14-1 PE**	677.42	$[M-3H]^-$
**17:0-16:1 PE**	705.39	$[M-3H]^-$
**17:0-18:1 PE**	733.37	$[M-3H]^-$
**17:0-20:3 PE**	757.34	$[M-3H]^-$
**17:0-22:4 PE**	783.34	$[M-3H]^-$
**adonitol**	152.36	$[M]^{\bullet -}$

**alanine**	90.65	$[M]^{\bullet -}$
**fructose**	180.01	$[M]^{\bullet -}$
**glucose**	180.21	$[M]^{\bullet -}$
**palmitic acid**	255.41	$[M]^{\bullet -}$
**oleic acid**	281.31	$[M]^{\bullet -}$
**phenylalanine**	165.44	$[M]^{\bullet -}$
**serine**	106.07	$[M]^{\bullet -}$
**shikimic acid**	173.94	$[M]^{\bullet -}$
plate AgPs0.03		
**14:0-13:0-14:0 TG**	736.03	$[M+Na]^+$
**14:0-15:1-14:0 TG**	846.74	$[M+^{107}Ag]^+$
**14:0-17:1-14:0 TG**	790.90	$[M+Na]^+$
**16:0-15:1-16:0 TG**	818.82	$[M+Na]^+$
**16:0-17:1-16:0 TG**	846.74	$[M+Na]^+$
**16:0-19:2-16:0 TG**	872.65	$[M+Na]^+$
**18:1-17:1-18:1 TG**	898.89	$[M+Na]^+$
**18:1-19:2-18:1 TG**	924.44	$[M+Na]^+$
**18:1-21:2-18:1 TG**	968.54	$[M+K]^+$
**adonitol**	258.98	$[M+^{107}Ag]^+$
**cholesterol**	493.38	$[M+^{107}Ag]^+$
**alanine**	195.96	$[M+^{107}Ag]^+$
**fructose**	286.98	$[M+^{107}Ag]^+$
**glucose**	286.97	$[M+^{107}Ag]^+$
**methionine**	255.92	$[M+^{107}Ag]^+$
**palmitic acid**	363.13	$[M+^{107}Ag]^+$
**oleic acid**	389.15	$[M+^{107}Ag]^+$
**phenylalanine**	271.99	$[M+^{107}Ag]^+$
**serine**	211.91	$[M+^{107}Ag]^+$
**shikimic acid**	280.97	$[M+^{107}Ag]^+$
**17:0-14-1 PE**	677.75	$[M-3H]^-$
**17:0-16:1 PE**	705.76	$[M-3H]^-$
**17:0-18:1 PE**	733.80	$[M-3H]^-$
**17:0-20:3 PE**	757.83	$[M-3H]^-$
**17:0-22:4 PE**	783.88	$[M-3H]^-$
**adonitol**	152.34	$[M]^{\bullet -}$

**cholesterol**	385.83	$[M]^{\bullet-}$
**alanine**	89.97	$[M]^{\bullet-}$
**fructose**	180.01	$[M]^{\bullet-}$
**glucose**	180.29	$[M]^{\bullet-}$
**methionine**	149.63	$[M]^{\bullet-}$
**palmitic acid**	256.58	$[M]^{\bullet-}$
**oleic acid**	281.31	$[M]^{\bullet-}$
**phenylalanine**	165.46	$[M]^{\bullet-}$
**serine**	105.96	$[M]^{\bullet-}$
**shikimic acid**	174.00	$[M]^{\bullet-}$
plate AgPs0.04		
**14:0-13:0-14:0 TG**	736.20	$[M+Na]^{+}$
**14:0-15:1-14:0 TG**	762.11	$[M+Na]^{+}$
**14:0-17:1-14:0 TG**	790.00	$[M+Na]^{+}$
**16:0-15:1-16:0 TG**	818.87	$[M+Na]^{+}$
**16:0-17:1-16:0 TG**	846.73	$[M+Na]^{+}$
**16:0-19:2-16:0 TG**	872.56	$[M+Na]^{+}$
**18:1-17:1-18:1 TG**	898.38	$[M+Na]^{+}$
**18:1-19:2-18:1 TG**	924.17	$[M+Na]^{+}$
**18:1-21:2-18:1 TG**	952.81	$[M+H]^{+}$
**adonitol**	258.97	$[M+^{107}Ag]^{+}$
**cholesterol**	493.28	$[M+^{107}Ag]^{+}$
**alanine**	195.97	$[M+^{107}Ag]^{+}$
**fructose**	286.96	$[M+^{107}Ag]^{+}$
**glucose**	286.97	$[M+^{107}Ag]^{+}$
**methionine**	255.98	$[M+^{107}Ag]^{+}$
**palmitic acid**	363.44	$[M+^{107}Ag]^{+}$
**oleic acid**	389.09	$[M+^{107}Ag]^{+}$
**phenylalanine**	272.00	$[M+^{107}Ag]^{+}$
**serine**	211.83	$[M+^{107}Ag]^{+}$
**shikimic acid**	280.97	$[M+^{107}Ag]^{+}$
**15:0 LPC**	487.12	$[M+H]^{+}$
**17:0 LPC**	515.47	$[M+H]^{+}$
**19:0 LPC**	543.01	$[M+H]^{+}$
**adonitol**	152.24	$[M]^{\bullet-}$

**cholesterol**	385.83	$[M]^{\bullet-}$
**alanine**	90.16	$[M]^{\bullet-}$
**fructose**	180.36	$[M]^{\bullet-}$
**glucose**	180.32	$[M]^{\bullet-}$
**methionine**	149.57	$[M]^{\bullet-}$
**palmitic acid**	255.51	$[M]^{\bullet-}$
**oleic acid**	281.38	$[M]^{\bullet-}$
**phenylalanine**	165.41	$[M]^{\bullet-}$
**serine**	106.01	$[M]^{\bullet-}$
**shikimic acid**	173.96	$[M]^{\bullet-}$
plate AgPs0.06		
**14:0-13:0-14:0 TG**	736.24	$[M+Na]^+$
**14:0-15:1-14:0 TG**	846.78	$[M+^{107}Ag]^+$
**14:0-17:1-14:0 TG**	790.05	$[M+Na]^+$
**16:0-15:1-16:0 TG**	818.93	$[M+Na]^+$
**16:0-17:1-16:0 TG**	846.78	$[M+Na]^+$
**16:0-19:2-16:0 TG**	872.62	$[M+Na]^+$
**18:1-17:1-18:1 TG**	898.44	$[M+Na]^+$
**18:1-19:2-18:1 TG**	924.25	$[M+Na]^+$
**18:1-21:2-18:1 TG**	952.02	$[M+Na]^+$
**adonitol**	258.97	$[M+^{107}Ag]^+$
**cholesterol**	493.65	$[M+^{107}Ag]^+$
**alanine**	195.96	$[M+^{107}Ag]^+$
**fructose**	286.97	$[M+^{107}Ag]^+$
**glucose**	286.96	$[M+^{107}Ag]^+$
**methionine**	255.96	$[M+^{107}Ag]^+$
**palmitic acid**	363.45	$[M+^{107}Ag]^+$
**oleic acid**	389.03	$[M+^{107}Ag]^+$
**phenylalanine**	271.99	$[M+^{107}Ag]^+$
**serine**	211.97	$[M+^{107}Ag]^+$
**shikimic acid**	280.95	$[M+^{107}Ag]^+$
**adonitol**	152.36	$[M]^{\bullet-}$
**cholesterol**	385.82	$[M]^{\bullet-}$
**alanine**	90.34	$[M]^{\bullet-}$
**fructose**	180.11	$[M]^{\bullet-}$

**glucose**	180.15	$[M]^{\bullet-}$
**methionine**	149.42	$[M]^{\bullet-}$
**palmitic acid**	255.41	$[M]^{\bullet-}$
**oleic acid**	281.03	$[M]^{\bullet-}$
**phenylalanine**	165.46	$[M]^{\bullet-}$
**serine**	106.04	$[M]^{\bullet-}$
**shikimic acid**	174.07	$[M]^{\bullet-}$
plate AgPs0.11		
**14:0-13:0-14:0 TG**	736.71	$[M+Na]^{+}$
**14:0-15:1-14:0 TG**	762.72	$[M+Na]^{+}$
**14:0-17:1-14:0 TG**	790.75	$[M+Na]^{+}$
**16:0-15:1-16:0 TG**	818.74	$[M+Na]^{+}$
**16:0-17:1-16:0 TG**	846.81	$[M+Na]^{+}$
**16:0-19:2-16:0 TG**	872.77	$[M+Na]^{+}$
**18:1-17:1-18:1 TG**	898.81	$[M+Na]^{+}$
**18:1-19:2-18:1 TG**	924.83	$[M+Na]^{+}$
**18:1-21:2-18:1 TG**	952.85	$[M+Na]^{+}$
**adonitol**	258.45	$[M+^{107}Ag]^{+}$
**cholesterol**	493.62	$[M+^{107}Ag]^{+}$
**alanine**	195.98	$[M+^{107}Ag]^{+}$
**fructose**	286.99	$[M+^{107}Ag]^{+}$
**glucose**	286.96	$[M+^{107}Ag]^{+}$
**methionine**	255.96	$[M+^{107}Ag]^{+}$
**palmitic acid**	363.11	$[M+^{107}Ag]^{+}$
**oleic acid**	389.35	$[M+^{107}Ag]^{+}$
**phenylalanine**	271.99	$[M+^{107}Ag]^{+}$
**serine**	211.81	$[M+^{107}Ag]^{+}$
**shikimic acid**	280.95	$[M+^{107}Ag]^{+}$
**adonitol**	152.38	$[M]^{\bullet-}$
**cholesterol**	385.43	$[M]^{\bullet-}$
**alanine**	89.78	$[M]^{\bullet-}$
**fructose**	180.24	$[M]^{\bullet-}$
**glucose**	180.20	$[M]^{\bullet-}$
**methionine**	149.40	$[M]^{\bullet-}$
**palmitic acid**	255.38	$[M]^{\bullet-}$

**oleic acid**	281.29	$[M]^{\bullet-}$
**phenylalanine**	165.22	$[M]^{\bullet-}$
**serine**	105.27	$[M]^{\bullet-}$
**shikimic acid**	174.06	$[M]^{\bullet-}$
**17:0-14-1 PE**	678.25	$[M-2H]^{-}$
**17:0-16:1 PE**	706.24	$[M-2H]^{-}$
**17:0-18:1 PE**	734.24	$[M-2H]^{-}$
**17:0-20:3 PE**	758.23	$[M-2H]^{-}$
**17:0-22:4 PE**	784.25	$[M-2H]^{-}$
**14:0-13:0-14:0 TG**	711.23	$[M-2H]^{-}$
**14:0-15:1-14:0 TG**	737.23	$[M-2H]^{-}$
**14:0-17:1-14:0 TG**	765.26	$[M-2H]^{-}$
**16:0-15:1-16:0 TG**	793.29	$[M-2H]^{-}$
**16:0-17:1-16:0 TG**	821.34	$[M-2H]^{-}$
**16:0-19:2-16:0 TG**	847.38	$[M-2H]^{-}$
**18:1-17:1-18:1 TG**	873.42	$[M-2H]^{-}$
**18:1-19:2-18:1 TG**	899.48	$[M-2H]^{-}$
plate AgPs0.17		
**14:0-15:1-14:0 TG**	846.35	$[M+^{107}Ag]^{+}$
**14:0-17:1-14:0 TG**	874.26	$[M+^{107}Ag]^{+}$
**16:0-15:1-16:0 TG**	818.27	$[M+Na]^{+}$
**16:0-17:1-16:0 TG**	846.72	$[M+Na]^{+}$
**16:0-19:2-16:0 TG**	872.42	$[M+Na]^{+}$
**18:1-17:1-18:1 TG**	898.53	$[M+Na]^{+}$
**18:1-19:2-18:1 TG**	902.38	$[M+H]^{+}$
**18:1-21:2-18:1 TG**	930.55	$[M+H]^{+}$
**adonitol**	259.00	$[M+^{107}Ag]^{+}$
**cholesterol**	493.20	$[M+^{107}Ag]^{+}$
**alanine**	195.96	$[M+^{107}Ag]^{+}$
**fructose**	286.99	$[M+^{107}Ag]^{+}$
**glucose**	286.99	$[M+^{107}Ag]^{+}$
**methionine**	255.98	$[M+^{107}Ag]^{+}$
**palmitic acid**	363.14	$[M+^{107}Ag]^{+}$
**oleic acid**	389.06	$[M+^{107}Ag]^{+}$
**phenylalanine**	272.01	$[M+^{107}Ag]^{+}$

**serine**	211.76	$[M+^{107}Ag]^+$
**shikimic acid**	280.99	$[M+^{107}Ag]^+$
**adonitol**	152.54	$[M]^{\bullet-}$
**cholesterol**	385.79	$[M]^{\bullet-}$
**alanine**	90.46	$[M]^{\bullet-}$
**fructose**	180.14	$[M]^{\bullet-}$
**glucose**	180.11	$[M]^{\bullet-}$
**methionine**	149.55	$[M]^{\bullet-}$
**palmitic acid**	255.37	$[M]^{\bullet-}$
**oleic acid**	281.30	$[M]^{\bullet-}$
**phenylalanine**	165.39	$[M]^{\bullet-}$
**serine**	106.20	$[M]^{\bullet-}$
**shikimic acid**	174.07	$[M]^{\bullet-}$
plate AgPs0.19		
**14:0-13:0-14:0 TG**	736.63	$[M+Na]^+$
**14:0-15:1-14:0 TG**	846.76	$[M+^{107}Ag]^+$
**14:0-17:1-14:0 TG**	790.69	$[M+Na]^+$
**16:0-15:1-16:0 TG**	818.72	$[M+Na]^+$
**16:0-17:1-16:0 TG**	846.76	$[M+Na]^+$
**16:0-19:2-16:0 TG**	872.78	$[M+Na]^+$
**18:1-17:1-18:1 TG**	898.79	$[M+Na]^+$
**18:1-19:2-18:1 TG**	924.81	$[M+Na]^+$
**18:1-21:2-18:1 TG**	952.85	$[M+Na]^+$
**adonitol**	259.00	$[M+^{107}Ag]^+$
**cholesterol**	493.22	$[M+^{107}Ag]^+$
**alanine**	195.77	$[M+^{107}Ag]^+$
**fructose**	286.98	$[M+^{107}Ag]^+$
**glucose**	286.98	$[M+^{107}Ag]^+$
**methionine**	255.97	$[M+^{107}Ag]^+$
**palmitic acid**	363.12	$[M+^{107}Ag]^+$
**oleic acid**	389.05	$[M+^{107}Ag]^+$
**phenylalanine**	271.98	$[M+^{107}Ag]^+$
**serine**	211.80	$[M+^{107}Ag]^+$
**shikimic acid**	280.95	$[M+^{107}Ag]^+$
**adonitol**	152.39	$[M]^{\bullet-}$

**alanine**	90.15	$[M]^{\bullet -}$
**fructose**	180.03	$[M]^{\bullet -}$
**glucose**	180.41	$[M]^{\bullet -}$
**methionine**	149.55	$[M]^{\bullet -}$
**palmitic acid**	255.44	$[M]^{\bullet -}$
**oleic acid**	281.32	$[M]^{\bullet -}$
**phenylalanine**	165.41	$[M]^{\bullet -}$
**serine**	106.17	$[M]^{\bullet -}$
**shikimic acid**	174.51	$[M]^{\bullet -}$
plate AgPs0.2		
**14:0-13:0-14:0 TG**	736.66	$[M+Na]^+$
**14:0-15:1-14:0 TG**	762.68	$[M+Na]^+$
**14:0-17:1-14:0 TG**	790.71	$[M+Na]^+$
**16:0-15:1-16:0 TG**	818.74	$[M+Na]^+$
**16:0-17:1-16:0 TG**	846.77	$[M+Na]^+$
**16:0-19:2-16:0 TG**	872.78	$[M+Na]^+$
**18:1-17:1-18:1 TG**	898.80	$[M+Na]^+$
**18:1-19:2-18:1 TG**	924.81	$[M+Na]^+$
**18:1-21:2-18:1 TG**	952.83	$[M+Na]^+$
**adonitol**	258.99	$[M+^{107}Ag]^+$
**cholesterol**	493.36	$[M+^{107}Ag]^+$
**alanine**	195.95	$[M+^{107}Ag]^+$
**fructose**	286.99	$[M+^{107}Ag]^+$
**glucose**	286.99	$[M+^{107}Ag]^+$
**methionine**	255.97	$[M+^{107}Ag]^+$
**palmitic acid**	363.16	$[M+^{107}Ag]^+$
**oleic acid**	389.11	$[M+^{107}Ag]^+$
**phenylalanine**	272.00	$[M+^{107}Ag]^+$
**serine**	211.76	$[M+^{107}Ag]^+$
**shikimic acid**	280.99	$[M+^{107}Ag]^+$
**adonitol**	152.30	$[M]^{\bullet -}$
**alanine**	90.36	$[M]^{\bullet -}$
**fructose**	180.45	$[M]^{\bullet -}$
**glucose**	180.41	$[M]^{\bullet -}$
**methionine**	149.36	$[M]^{\bullet -}$

**palmitic acid**	255.42	$[M]^{\bullet-}$
**oleic acid**	281.33	$[M]^{\bullet-}$
**phenylalanine**	165.15	$[M]^{\bullet-}$
**serine**	106.08	$[M]^{\bullet-}$
**shikimic acid**	174.45	$[M]^{\bullet-}$
**17:0-14-1 PE**	677.90	$[M\text{-}3H]^{-}$
**17:0-16:1 PE**	705.93	$[M\text{-}3H]^{-}$
**17:0-18:1 PE**	733.97	$[M\text{-}3H]^{-}$
**17:0-20:3 PE**	757.99	$[M\text{-}2H]^{-}$
**17:0-22:4 PE**	784.04	$[M\text{-}2H]^{-}$
